# Cholecystokinin 1 Receptor – A Unique *G P*rotein-*C*oupled *R*eceptor *A*ctivated *b*y *S*inglet *O*xygen (*GPCR-ABSO*)

**DOI:** 10.3389/fphys.2018.00497

**Published:** 2018-05-08

**Authors:** Hong Ning Jiang, Yuan Li, Wen Yi Jiang, Zong Jie Cui

**Affiliations:** Institute of Cell Biology, Beijing Normal University, Beijing, China

**Keywords:** photodynamic actuation, photodynamic physiology, photosensitization, *G p*rotein *c*oupled *r*eceptor *a*ctivated *b*y *s*inglet *o*xygen, *G p*rotein *c*oupled *r*eceptor *a*ctivated *b*y *p*hotodynamic *a*ction

## Abstract

Plasma membrane-delimited generation of singlet oxygen by photodynamic action with photosensitizer sulfonated aluminum phthalocyanine (SALPC) activates cholecystokinin 1 receptor (CCK1R) in pancreatic acini. Whether CCK1R retains such photooxidative singlet oxygen activation properties in other environments is not known. Genetically encoded protein photosensitizers KillerRed or mini singlet oxygen generator (miniSOG) were expressed in pancreatic acinar tumor cell line AR4-2J, CCK1R, KillerRed or miniSOG were expressed in HEK293 or CHO-K1 cells. Cold light irradiation (87 mW⋅cm^-2^) was applied to photosensitizer-expressing cells to examine photodynamic activation of CCK1R by Fura-2 fluorescent calcium imaging. When CCK1R was transduced into HEK293 cells which lack endogenous CCK1R, photodynamic action with SALPC was found to activate CCK1R in CCK1R-HEK293 cells. When KillerRed or miniSOG were transduced into AR4-2J which expresses endogenous CCK1R, KillerRed or miniSOG photodynamic action at the plasma membrane also activated CCK1R. When fused KillerRed-CCK1R was transduced into CHO-K1 cells, light irradiation activated the fused CCK1R leading to calcium oscillations. Therefore KillerRed either expressed independently, or fused with CCK1R can both activate CCK1R photodynamically. It is concluded that photodynamic singlet oxygen activation is an intrinsic property of CCK1R, independent of photosensitizer used, or CCK1R-expressing cell types. Photodynamic singlet oxygen CCK1R activation after transduction of genetically encoded photosensitizer *in situ* may provide a convenient way to verify intrinsic physiological functions of CCK1R in multiple CCK1R-expressing cells and tissues, or to actuate CCK1R function in CCK1R-expressing and non-expressing cell types after transduction with fused KillerRed-CCK1R.

## Introduction

The delta singlet oxygen (^1^Δ_g_; referred to hereafter as singlet oxygen or ^1^O_2_) generated in a type II photodynamic action reacts with cellular components to trigger different cellular responses (Cui and Matthews, 1998; Cui et al., 2012; Jiang et al., 2017). Concentrated photodynamic ^1^O_2_ generation is cytocidal – at mitochondria and lysosomes leading to apoptosis, at the ER autophagy, and at the plasma membrane necrosis (Bacellar et al., 2015; Abrahamse and Hamblin, 2016). Photodynamic therapy is therefore useful to treat both cancer and other lesions (Agostinis et al., 2011; Huggett et al., 2014; Craig et al., 2015; Liu et al., 2016; Newman, 2016). Controlled photodynamic generation of ^1^O_2_ at lower doses, however, has been found to modulate specific cellular signaling pathways (Cui and Matthews, 1998; Cui et al., 2012; Krammer and Verwanger, 2012; Bacellar et al., 2015). One particular photodynamic ^1^O_2_ target is the cholecystokinin 1 (CCK1) receptor.

We have unambiguously confirmed permanent photodynamic activation of CCK1 receptor (CCK1R) and the associated amylase secretion in pancreatic acinar cells (Matthews and Cui, 1989, 1990a,b; al-Laith et al., 1993; Cui and Kanno, 1997; Cui et al., 1997, 2012; An et al., 2003). In such experiments, freshly isolated pancreatic acini were perifused, briefly exposed (10 min) to photosensitizer sulphonated aluminum phthalocyanine (SALPC), before washing out unbound SALPC. Subsequent light illumination (1 min) of rat pancreatic acini with plasma membrane-bound SALPC then triggered persistent calcium oscillations, in sharp contrast with oscillations induced by physiological CCK concentrations, which disappeared immediately after CCK wash-out (Cui and Kanno, 1997; Cui et al., 1997). Photodynamically induced calcium oscillations were blocked by CCK1R antagonist FK480; after FK480 blockade of photodynamic calcium oscillations, the muscarinic agonist bethanechol triggered new calcium oscillations, indicating that after permanent photodynamic CCK1R activation and subsequent CCK1R blockade with FK480, pancreatic acinar cells *remained perfectly healthy* (An et al., 2003; Cui et al., 2012).

^1^O_2_ in the cellular *milieu* has a short lifetime (μs; Cui and Matthews, 1998; Bovis et al., 2012; Kim et al., 2014), and therefore has a limited effective diffusion or reactive distance (<10 nm; Moan and Berg, 1991; Cui and Matthews, 1998; Dougherty et al., 1998; Nowis et al., 2005; Cui et al., 2012). ^1^O_2_ generated in photodynamic action is therefore effective only at the site of generation, i.e., at the site of photosensitizer. Although in our previous experiments photosensitizer SALPC was incubated with pancreatic acini briefly to limit SALPC-binding to plasma membrane, therefore limiting ^1^O_2_ generation to plasma membrane, ^1^O_2_ localization would be much improved if the photosensitizer could be targeted with higher specificity and precision. Genetically encoded protein photosensitizers, due to the possibility of fusion with signal sequences for specific subcellular targeting, would be perfect for such precise localization.

Genetically encoded KillerRed or miniSOG target-expressed with signal sequence tags at specific subcellular organelles (Qi et al., 2012; Ryumina et al., 2013; Jarvela and Linstedt, 2014; Serebrovskaya et al., 2014) or by fusion-expression with target proteins (Serebrovskaya et al., 2011; Lin et al., 2013; Waldeck et al., 2013; Zhou et al., 2013; Sun et al., 2015) in specific cell types under tissue-specific promoters (Lin et al., 2013; Zhou et al., 2013) have been shown to be highly efficient precisely localized photosensitizers. Such cellular organelle-delimited protein photosensitizers after light irradiation would generate ^1^O_2_ locally in spatially defined fashion and *photooxidize* nanoscopically (<10 nm) target proteins such as plasma membrane CCK1R.

The pancreatic acinar cells are typical CCK1R-expressing cells vital for digestive enzyme secretion (Cui and Kanno, 1997; Cui et al., 1997; An et al., 2003; Liang et al., 2013, 2017). But it has not been demonstrated whether CCK1R expressed in other cell types would be equally susceptible to photodynamic ^1^O_2_ activation. Therefore the aims of the present work were: (i) to examine whether CCK1R ectopically expressed in cells other than pancreatic acinar cells could be activated by SALPC photodynamic action, and (ii) to examine whether photodynamic action with KillerRed or miniSOG target-expressed to the plasma membrane could activate photodynamically the CCK1R in CCK1R-expressing pancreatic acinar tumor cell line AR4-2J, and in cell lines ectopically expressing CCK1R. The present work confirmed that CCK1R is photodynamically activated irrespective of the photosensitizers used or cell types where CCK1R is expressed. Importantly, KillerRed fused with CCK1R retains its photodynamic effect to activate the fused CCK1R after light irradiation. This important new finding may immediately open up new avenues to elucidate CCK1R physiology and extend CCK1R pharmacology *in vivo* both in the central nervous system and in peripheral organs.

## Materials and Methods

### Materials

Sulfated cholecystokinin octopeptide (CCK), CCK1R antagonist devazepide were from Tocris Cookson (Bristol, United Kingdom). MEM amino acid mixture (50×), DMEM/F12, penicillin/streptomycin, Opti-MEM, and MitoTracker^®^ Green FM were from InVitrogen (Shanghai, China). 4-(2-Hydroxyethyl)-1-piperazine-ethane-sulfonic acid (HEPES) was from Calbiochem (Darmstadt, Germany). Fura-2 AM was from AAT Bioquest (Sunnyvale, CA, United States). Transfection reagent X-tremeGENE HP was from Roche (Mannheim, Germany). Cell-Tak and Agar (Bacto^TM^) were from BD Biosciences (Bedford, MA, United States). Fetal bovine serum (FBS) was from Thermo Scientific (Shanghai, China). pKillerRed_mem_ and pKillerRed_dMito_ were bought from Evrogen (Moscow, Russia). Yeast extract and tryptone were from MERCK (Darmstadt, Germany). Endotoxin-free plasmid extraction kit and DH5à competent cells were from TianGen Biochemicals (Beijing, China). Restriction enzymes (BamHI, EcoRI, and XhoI) were from Takara (Beijing, China). Goat and rabbit anti-CCK1R polyclonal antibody, 2nd ab (FITC- or DyLight 488-labeled) were from Abcam (Cambridge, United Kingdom). Hoechst 33342 was from DojinDo (Beijing, China). Photosensitizer SALPC was from Frontier Scientific (AlPcS-834, Logan, UT, United States).

### Cell Culture (AR4-2J, HEK293, CHO-K1, *E. coli*)

AR4-2J was bought from ATCC (Rockville, MD, United States) and cultured in DMEM/F12 supplemented with 20% fetal bovine serum (Hyclone) and antibiotics in a CO_2_ incubator under 5% CO_2_ at 37°C as before (Matthews and Cui, 1990a,b; Cui, 1998; Duan et al., 2011; Liang et al., 2013). HEK293 and CHO-K1 were purchased from Shanghai Institutes of Life Sciences Chinese Academy of Sciences and cultured in DMEM/F12 similarly.

Solid *E. coli* medium LB/Kana was sterilized and culture plates made. Liquid *E. coli* medium LB/Kana had the same composition but without agar.

### Immunocytochemistry

Dispersed cells were attached to Cell-Tak-coated cover-slips before being fixed in paraformaldehyde 4% (10 min). Cells were permeabilized in 0.2% Triton X. Non-specific binding was blocked in 3% BSA (PBS) before incubation with primary antibodies in a humid chamber at 4°C overnight, followed by incubation with 2nd ab (30 min). Cover-slips were placed on a slide, sealed and stored at 4°C. For double staining, incubation with 1st Ab was repeated, and incubation with 2nd Ab was performed with mixed 2nd Abs. Imaging was done in a confocal microscope (Zeiss LSM 510 META, objective 63 × /1.40 oil).

### Vector Constructs

Plasmids pKillerRed_mem_ and pKillerRed_dmito_ were bought from Evrogen (Moscow, Russia). Competent *E. coli* were infected with plasmid, cultured on solid LB/Kana. Bacteria colonies were picked and further cultured in liquid LB/Kana with shaking overnight. Proliferated plasmid was extracted and sequence verified.

pKillerRed_lyso_ was constructed fusing pKillerRed_mem_ with lysosomal localization sequence KGQGSMDEGTADERAPLIRT via in-fusion cloning. Forward primer 5′-CGCGGGCCCGGGATCCATGAAAGGACAGGGATCCATGGATGAGGGAACAGCGGATGAAAGAGCACCCCTCATTCGAACCTCCGAGGGCGGCCCCG-3′ was annealed with reverse primer 5′-CGGGGCCGCCCTCGGAGGTTCGAATGAGGGGTGCTCTTTCATCCGCTGTTCCCTCATCCATGGATCCCTGTCCTTTCATGGATCCCGGGCCCGCG-3′ to obtain a double stranded complementary DNA. pKillerRed_mem_ was linearized after digestion with BamH1-specific endonuclease. The above double stranded complementary DNA was fused with linearized pKillerRed_mem_ by a fusion HD enzyme, to obtain vector pKillerRed_lyso_. pKillerRed_lyso_ was transformed into DH5α, harvested and sequenced for verification.

To construct vector CCK1R-pKillerRed plasmid pKillerRed_mem_ was used as template to amplify KillerRed by PCR. The forward primer was 5′-GGATCCATGCTGTGCTGTATGAGAAGAA-3′, downstream primer was 3′-GAATTCATCCTCGTCGCTACCGATG-5′. The forward primer contained a BamHI site, the reverse an EcoRI site. These two restrictive sites were used to cut the PCR products and pcDNA3.1-CCK1R and target fragments were cloned into pcDNA3.1-CCK1R by transforming DH5α. The recombinant plasmids were extracted and the sequence verified and named CCK1R-pKillerRed. The transduced cells were named CCK1R-pKillerRed-CHO-K1.

The miniSOG sequence was synthesized and inserted into pKillerRed_mem_ to replace the KillerRed sequence. A mammalian codon-optimized miniSOG gene (GenBank accession number JX999997) was assembled from oligonucleotides by gene synthesis (Genscript, Nanjing, China). The miniSOG sequence used was ATGGAAAAGAGCTTTGTGATTACCGATCCGCGCCTGCCAGACAACCCGATCATTTTCGCGAGCGATGGCTTTCTGGAGTTAACCGAATATTCTCGTGAGGAAATTCTGGGTCGCAATGGCCGTTTCTTGCAGGGTCCGGAAACGGATCAAGCCACCGTGCAGAAAATCCGCGATGCGATTCGTGACCAACGCGAAATCACCGTTCAGCTGATTAACTATACGAAAAGCGGCAAGAAATTTTGGAACTTACTGCATCTGCAACCGATGCGCGATCAGAAAGGCGAATTGCAATATTTCATTGGTGTGCAGCTGGATGGCTAG. The plasmid was named pminiSOG_mem_.

### Transduction of HEK293, AR4-2J, and CHO-K1 Cells

HEK293, AR4-2J, CHO-K1 cells at 80% confluence were dispersed and planted in 6-well plates, transfected 24 h later in Opti-MEM medium containing plasmid and transfection reagent (X-tremeGENE HP or Lipofectamine 2000).

Plasmid miniSOG_mem_ was transduced into AR4-2J by electroporation (250 V 1500 mF 150 Ω 30 ms) in a Gene Pulser (MXcell, BIO-RAD, CA, United States). AR4-2J suspension containing 20 μg/mL pminiSOG_mem_ DNA was used for electroporation. Cells were re-suspended in complete medium immediately after electroporation, plated on coverslips, and used 24–48 h later.

To load transduced cells with fluorescent probes for mitochondrial or lysosomal tracking, cells were incubated with MitoTracker Green (0.05 mM) or LysoTracker Green (0.075 mM) for 30 min before imaging.

### Photodynamic Action

To trigger photodynamic action, KillerRed- or miniSOG-expressing cells were irradiated with white light (87 mW⋅cm^-2^), SALPC-bound cells were irradiated with red light (>580 nm, 36.7 mW⋅cm^-2^), from a halogen cold light source (MegaLight 100, Hoya-Schott, Japan). Illuminance and irradiance were measured with a power meter (IL1700, International Light Inc., Newburyport, MA, United States). The fluorescence emission lightpath (for calcium measurements) need not be deflected off the detector during red light irradiation since Fura-2 emission was cut off at 550 nm (emitter D510/40 nm, see below).

### Calcium Measurements

Dispersed cells were loaded with Fura-2 AM (10 μM, 30 min), attached to Cell-Tak-coated cover-slip bottom of Sykes-Moore chambers for 30 min before perfusion. Cytosolic calcium was measured in an inverted fluorescent microscope (Olympus IX 70 or Nikon NE3000) coupled to a calcium measurement system (PTI, New Jersey, United States) with alternating excitations at 340 nm/380 nm (DeltaRam V or X). Emission (dichroic mirror 400DCLP, emitter D510/40 nm) was detected with a PMT (pmt814, PTI) or a CCD (NEO-5.5-CL-3, Andor). Calcium concentration was expressed as F_340_/F_380_ and plotted against time with SigmaPlot as reported before (Jia and Cui, 2011; Liang et al., 2013; Li et al., 2015).

### Statistical Analysis

To analyze the statistical significance of the differences of peak values before, during, and after devazepide (1 nM) in **Figure 3C**, all calcium peaks were normalized to the mean of peaks before perfusion of devazepide. Student’s *t-*test was used, and *P* < 0.05 was taken as statistically significant, indicated with an asterisk (^∗^).

## Results

### SALPC Photodynamic Activation of CCK1R Expressed in HEK293 Cells

**FIGURE 1:**
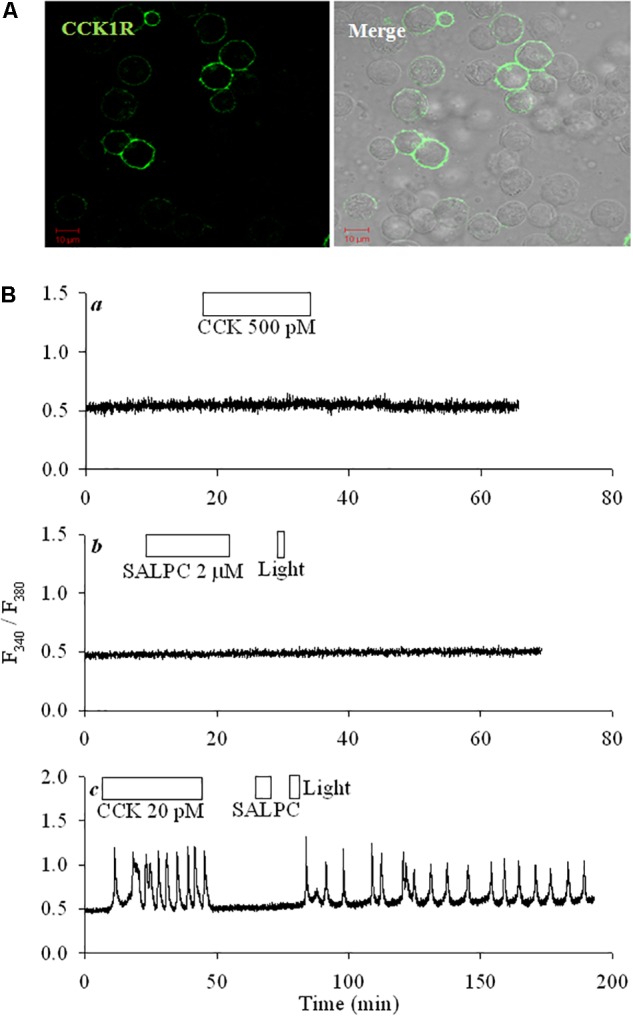
CCK1R ectopically expressed in HEK293 was activated by SALPC photodynamic action. **(A)** Fixed CCK1R-HEK293 cells (48 h after transfection) were attached to cover-slips, incubated sequentially with primary anti-CCK1R and DyLight 488-labeled 2nd Ab, imaged in a confocal microscope. Shown are fluorescent and merged bright field images. Controls (no primary and 2nd abs; with only 2nd Ab; primary Ab replaced with non-specific IgG) done in CCK1R-HEK293 cells did not show any fluorescence. Scale bars 10 μm. **(B)** Control non-transfected HEK293 cells **(Ba,b)** or CCK1R-HEK293 cells **(Bc)** were loaded with Fura-2 AM, attached to cover-slips, perifused, and exposed to CCK (20, 500 pM), SALPC (2 μM), red light (λ > 580 nm, 36.7 mW⋅cm^-2^) as indicated by the horizontal bars. Calcium traces shown are each representative of ≥3 independent experiments.

CCK1R was transiently transduced into HEK293 cells. Immunocytochemistry done 48 h after transfection showed plasma membrane CCK1R localization; note the complete lack of CCK1 receptor in un-transfected cells in the same field (**Figure 1A**). CCK up to 500 pM had no effect on cytosolic calcium in un-transfected cells (**Figure 1Ba**), in these un-transfected cells no changes in basal calcium were ever found after photodynamic action (SALPC 2 μM, light λ > 580 nm, 36.7 mW⋅cm^-2^; **Figure 1Bb**). CCK (20 pM) triggered regular calcium oscillations in CCK1R-HEK293 cells, CCK wash-out led to immediate cessation of induced calcium spikes (**Figure 1Bc**). Exposure to the chemical photosensitizer SALPC (2 μM) in the dark had no effect on basal calcium in the same cell (**Figure 1Bc**), but subsequent light irradiation (λ > 580 nm, 36.7 mW⋅cm^-2^) triggered regular and persistent calcium oscillations, which continued long after cessation of light illumination (**Figure 1Bc**). Note the difference of CCK- and photodynamically induced calcium oscillations: the former disappeared immediate after CCK wash-out, the latter continued long after completion of light irradiation.

To examine photodynamic CCK1 receptor activation with protein photosensitizers, KillerRed or miniSOG was transduced into pancreatic acinar tumor cell AR4-2J.

### KillerRed_mem_ Photodynamic Endogenous CCK1 Receptor Activation in AR4-2J Cells

**FIGURE 2:**
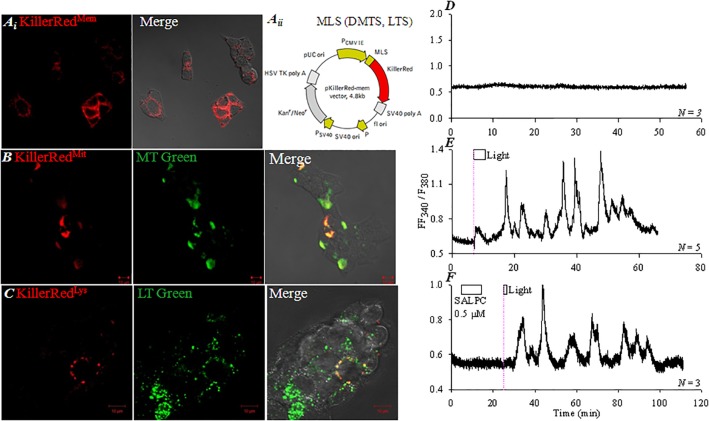
Targeted KillerRed expression and photodynamic CCK1R activation in KillerRed_mem_-AR4-2J cells. pKillerRed_mem_, pKillerRed_dmito_ and pKillerRed_lyso_ were expressed in AR4-2J cells, confocal images were taken 48 h after transfection. **(Ai)** pKillerRed_mem_. **(Aii)** pKillerRed_mem_, pKillerRed_dmito_, pKillerRed_lyso_. MLS, plasma membrane-localizing sequence; DMTS, duplicated mitochondria-targeting sequence; LTS, lysosome-targeting sequence. **(B)** pKillerRed_dmito_, mitochondria were visualized with MitoTracker Green. **(C)** pKillerRed_lyso_, lysosomes were visualized with LysoTracker Green. Fluorescent, bright field and merged images were taken by confocal microscopy: KillerRed λ_ex_ 543 nm, MitoTracker Green/LysoTracker Green λ_ex_ 488 nm. KillerRed_mem_-AR4-2J **(D,E)** or control AR4-2J cells **(F)** were loaded with Fura-2 AM, perifused. SALPC (0.5 μM) and light were applied as indicated by the horizontal bars. **(D)** KillerRed_mem_-AR4-2J cells without light irradiation. **(E)** KillerRed_mem_-AR4-2J cells after white light irradiation (87 mW⋅cm^-2^). **(F)** Non-transfected AR4-2J cells exposed to SALPC (0.5 μM), followed by red light irradiation (>580 nm, 36.7 mW⋅cm^-2^, 90 s). The thin pink dashed lines indicate start of light irradiation. Calcium traces shown are each representative of *N* identical experiments.

KillerRed (λ_ex_ 585 nm, λ_em_ 610 nm) was transduced into AR4-2J cells with organelle-targeting vectors (pKillerRed_mem_, pKillerRed_dmito_, pKillerRed_lyso_). Even plasma membrane distribution of KillerRed was found in pKillerRed_mem_-AR4-2J cells (**Figure 2Ai**). Mitochondrial or lysosomal targeting of KillerRed was also accomplished (**Figure 2Aii**), as confirmed by mitochondrial and lysosomal visualizations with MitoTracker Green (**Figure 2B**) and LysoTracker Green respectively (**Figure 2C**).

The pKillerRed_mem_-AR4-2J cells showed a stable cytosolic calcium baseline in the dark (**Figure 2D**), but white light irradiation (87 mW⋅cm^-2^) induced oscillatory increases in cytosolic calcium, which persisted after completion of light irradiation (**Figure 2E**). In non-transfected AR4-2J, basal calcium remained stable, chemical photosensitizer SALPC (0.5 μM) was perfused briefly, subsequent red light irradiation (> 580 nm, 36.7 mW⋅cm^-2^) induced calcium oscillations (**Figure 2F**) which were similar to white light irradiation-induced calcium oscillations in pKillerRed_mem_-AR4-2J cells (**Figure 2E**). These data indicate that KillerRed_mem_ photodynamic action fully duplicates SALPC photodynamic action (Cui and Kanno, 1997; An et al., 2003), both KillerRed and SALPC photodynamically activate the plasma membrane CCK1 receptor.

**FIGURE 3:**
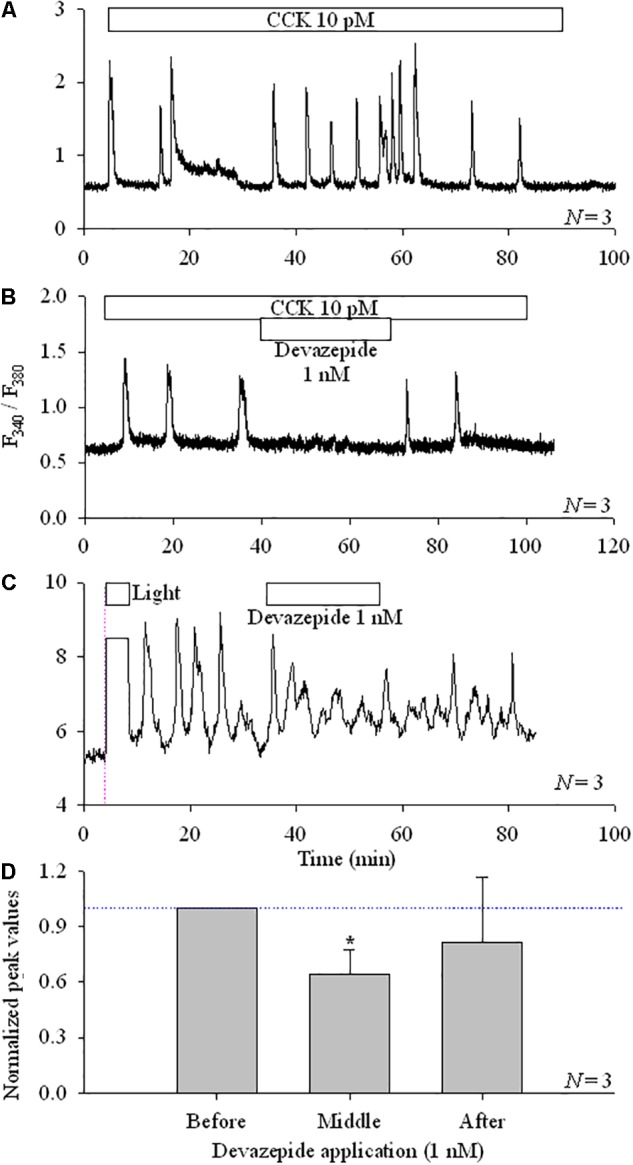
CCK1R antagonist devazepide inhibited photodynamically induced calcium oscillations in KillerRed_mem_-AR4-2J cells. Control, non-transfected AR4-2J **(A,B)** or KillerRed_mem_-AR4-2J cells **(C)** were loaded with Fura-2 AM and perifused. CCK (10 pM), devazepide (1 nM) and white light (87 mW⋅cm^-2^) were applied as indicated by the horizontal bars. Note that white light irradiation (87 mW⋅cm^-2^)-induced calcium oscillations in pKillerRed_mem_-AR4-2J cells were inhibited by devazepide. Vertical thin pink dashed line in **(C)** indicates start of light irradiation. Calcium traces are each representative of *N* identical experiments. **(D)** Experimental data as shown in **(C)** were analyzed, with all calcium peaks normalized to the mean of peaks before devazepide (taken as 1.00, indicated with a horizontal dashed blue line). The asterisk (^∗^) indicates statistical significance at *P* < 0.05. Note that in **(C)** the ordinate was different, due to the use of the CCD-based calcium measurement system. One only needs to note the dynamic changes.

CCK induced calcium oscillations in AR4-2J cells (**Figure 3A**), which were blocked by CCK1 antagonist devazepide (**Figure 3B**). KillerRed_mem_ photodynamic action-induced calcium oscillations were similarly inhibited by devazepide (**Figures 3C,D**).

### KillerRed_mem_ Retains Its Photodynamic Activating Effect of CCK1R After Fusion With CCK1R in CHO-K1 Cells

**FIGURE 4:**
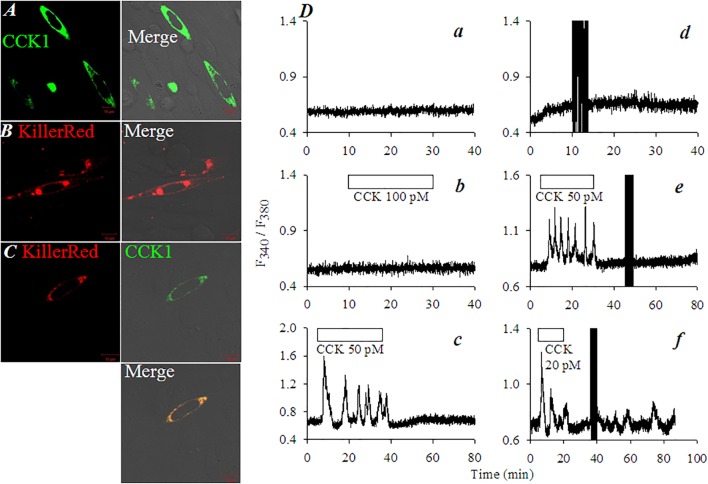
KillerRed_mem_ photodynamic activation of CCK1R in pCCK1R-KillerRed-CHO-K1 cells. KillerRed_mem_, KillerRed-CCK1R, CCK1R were expressed in CHO-K1 cells. At 48 h after transfection CHO-K1 cells were fixed for immunocytochemistry with primary Ab against CCK1R and FITC-tagged 2nd Ab **(A,C)**. KillerRed was visualized by intrinsic fluorescence **(B,C)**. **(A)** pCCK1R-CHO-K1 cells. **(B)** pKillerRed_mem_-CHO-K1 cells. **(C)** pKillerRed-CCK1R-CHO-K1 cells. Bright field, fluorescent, and merged images were obtained in a confocal microscope: KillerRed λ_ex_ 543 nm; FITC λ_ex_ 488 nm. Control non-transfected CHO-K1 cells**(Da,Db)**, pKillerRed_mem_-CHO-K1 cells **(Dd)**, pCCK1R-CHO-K1 cells **(De)**, or pCCK1R-KillerRed-CHO-K1 cells **(Dc,Df)** were loaded with Fura-2 AM, perifused, with CCK (20, 50, 100 pM) applied as indicated by the horizontal bars. The dark vertical bars (due to blockade of lightpath to detector during light irradiation) in **(Dd–Df)** indicate duration of white light irradiation (87 mW⋅cm^-2^). Calcium traces are each representative of three identical experiments.

To examine whether KillerRed retains its photodynamic CCK1R activating property when fused with CCK1R, vector pKillerRed-CCK1R was constructed. Both individual vectors (pKillerRed_mem_, pCCK1R) and recombinant vector pKillerRed-CCK1R (**Supplementary Figure S1**) were transduced into CHO-K1 cells which have no intrinsic CCK1R. CCK1R-CHO-K1 cells (**Figure 4A**), KillerRed_mem_-CHO-K1 cells (**Figure 4B**), and KillerRed-CCK1R-CHO-K1 cells (**Figure 4C**) expressed CCK1R, KillerRed, and KillerRed-CCK1R, respectively on plasma membrane (**Figures 4A–C**). Clear plasma membrane CCK1R/KillerRed co-localization was confirmed in pKillerRed-CCK1R-CHO-K1 cells (**Figure 4C**).

In un-transfected CHO-K1 cells the resting cytosolic calcium was stable (**Figure 4Da**). CHO-K1 cells do not express intrinsic CCK receptors; CCK 100 pM had no effect on basal calcium (**Figure 4Db**). In contrast, CCK 50 pM induced robust calcium oscillations in pCCK1R-CHO-K1 cells (**Figure 4Dc**). In pKillerRed_mem_-CHO-K1 cells (with KillerRed but not CCK1R transfections), white light irradiation (87 mW⋅cm^-2^) had no effect on basal calcium (**Figure 4Dd**). This indicates that *KillerRed photodynamic action had no effect on the essential calcium signaling machinery or on surface receptors other than CCK1 in CHO-1 cells*. CCK 50 pM induced strong calcium oscillations in pCCK1R-CHO-K1 cells; but white light irradiation (87 mW⋅cm^-2^) had no effect (**Figure 4De**). CCK 20 pM triggered calcium oscillations in pKillerRed-CCK1R-CHO-K1 cells as expected; wash-out of CCK led to immediate cessation of the induced calcium oscillations; subsequent white light irradiation (87 mW⋅cm^-2^) triggered fresh calcium oscillations (**Figure 4Df**). From these data it is clear that in pKillerRed-CCK1R-CHO-K1 cells, *both CCK1R and KillerRed retained their original activities*. KillerRed photodynamically activated the fused CCK1R to induce calcium oscillations.

### miniSOG_mem_ Photodynamic CCK1 Receptor Activation in AR4-2J Cells

**FIGURE 5:**
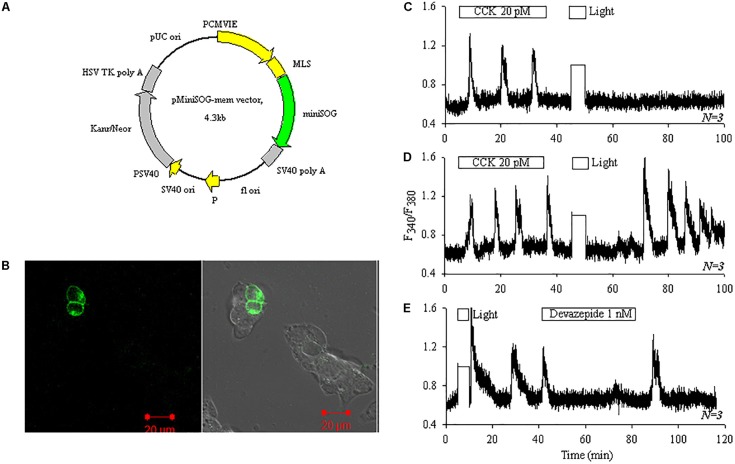
Photodynamic CCK1R activation triggers calcium oscillations in miniSOG_mem_-AR4-2J cells. AR4-2J cells were transfected with pminiSOG_mem_ **(A)**, and confocal imaged (λ_ex_ 488 nm). Note the lack of fluorescence in un-transfected cells. Fluorescent and bright field merged images are shown **(B)**. Control non-transfected AR4-2J cells (**C**, *N* = 3) or miniSOG_mem_-AR4-2J cells (**D**, *N* = 3; **E**, *N* = 3) were loaded with Fura-2 AM, and perifused. CCK (20 pM), devazepide (1 nM) and white light (87 mW⋅cm^-2^) were applied as indicated by the horizontal bars. Note the complete inhibition of calcium oscillations by CCK1 antagonist devazepide 1 nM in (**E**, *N* = 3). Calcium traces shown in **(C–E)** are each representative of *N* identical experiments. During white light irradiation in this figure both F340 and F380 were saturated (lightpath to detector not blocked) therefore the horizontal white bars in **(C–E)** also marked duration of light irradiation in the calcium traces.

miniSOG_mem_ (**Figure 5A**) was transduced into AR4-2J as demonstrated by confocal imaging (**Figure 5B**). CCK stimulation induced regular calcium oscillations and white light irradiation alone (87 mW⋅cm^-2^, 5 min) had no effect on basal calcium in non-transfected AR4-2J cells (**Figure 5C**). In miniSOG_mem_-AR4-2J cells, CCK induced reversible calcium oscillations but white light irradiation (87 mW⋅cm^-2^) induced persistent calcium oscillations (**Figure 5D**). Calcium oscillations induced by white light irradiation in miniSOG_mem_-AR4-2J cells were blocked completely by CCK1 antagonist devazepide 1 nM (**Figure 5E**).

## Discussion

In the present work we have found that CCK1R ectopically expressed in HEK293 was activated by SALPC photodynamic action, duplicating SALPC photodynamic CCK1R activation in isolated rat pancreatic acini. KillerRed_mem_ or miniSOG_mem_ photodynamically activated CCK1R in AR4-2J cells. Fused protein CCK1R-KillerRed_mem_ maintained both KillerRed and CCK1R activities, KillerRed_mem_ photodynamically activated CCK1R in KillerRed-CCK1R-CHO-K1 cells. These data together suggest that *photodynamic ^1^O_2_ activation is an intrinsic property of CCK1R*, independent of the photosensitizer used or cell types where CCK1R is expressed.

KillerRed has a GFP-like structure with a central chromophore of Q65-Y66-G67 (Pletnev et al., 2009; Roy et al., 2010). Q65-Y66-G67 connects with an aqueous channel (formed by I142, L143, P144, I199, I200, T201) to ensure oxygen supply from the medium. The excited chromophore transfers its excitation energy to ground state molecular oxygen to generate reactive oxygen species (ROS) which also exit KillerRed via this aqueous channel (Carpentier et al., 2009; Pletnev et al., 2009; Serebrovskaya et al., 2009; Roy et al., 2010). The purified KillerRed may undergo type I photodynamic action to generate superoxide (Pletnev et al., 2009; Shu et al., 2011; Vegh et al., 2011; Kim et al., 2014), although evidence for ^1^O_2_ generation by a Type II photodynamic action in the cellular context is very strong (Roy et al., 2010; Petrova et al., 2016). The case for ^1^O_2_ generation is indicated in the present work, since KillerRed photodynamically activated CCK1R, rather similar to SALPC photodynamic CCK1R activation. It is known that neither superoxide nor H_2_O_2_ had any effect on pancreatic acinar cell CCK1R (data not shown).

The flavin mononucleotide (FMN)-binding mini singlet oxygen generator (miniSOG) is composed of two α-helices interspersed in five β-sheets, with FMN located in between (Shu et al., 2011; Pietra, 2014). ^1^O_2_ probing with anthracene-9, 10-dipropionic acid (ADPA) obtained a quantum yield of 0.47 (Shu et al., 2011). But direct measurement of ^1^O_2_ phosphorescence at 1275 nm, and the use of uric acid as an ^1^O_2_ probe obtained a quantum yield of 0.03 (Ruiz-González et al., 2012; Pimenta et al., 2013). Since miniSOG is less than half the size of KillerRed, miniSOG may have some advantages over KillerRed in sensitizer/target protein fusion experiments, taking into account the fact that miniSOG_mem_ photodynamically activates CCK1R similarly.

The present work was carried out at the cellular level *in vitro*. Based on the present work, *in vivo* photodynamic ^1^O_2_ CCK1R activation is also possible. CCK1R activations *in situ* would have immediate physiological and pharmacological significance, either with peripheral or central CCK1R. CCK1R in nodose and dorsal root ganglia are known to play vital roles in satiety and other peripheral sensations (Broberger et al., 2001; Li et al., 2011; Kaczynska and Szereda-Przestaszewska, 2015). Highly localized CCK1R expression in the mouse hippocampus and defined extracortical sites are also well-recognized (Nishimura et al., 2015). Third ventricular ependymal cell CCK1R is known to be important for infant mouse satiety (Ozaki et al., 2013). A CCK-CCK receptor-like satiety-control system is commonly found in lower invertebrates such as *Caenorhabditis elegans* (Janssen et al., 2008; Bhattacharya et al., 2014). Our accumulated works suggest that CCK1R is unique among class A GPCR: it is activated permanently by type II photodynamic action (i.e., by ^1^O_2_). CCK1R is the only *G p*rotein-*c*oupled *r*eceptor *a*ctivated *b*y *s*inglet *o*xygen (^1^O_2_; *GPCR-ABSO*) identified so far, adding new arsenals alongside *RASSL* and *DREADD* (Alvarez-Curto et al., 2011; Gomez et al., 2017) for the elucidation of GPCR functions.

In conclusion, photodynamic activation is an intrinsic property of CCK1R, independent of photosensitizers used or CCK1R-expressing cell types. Photodynamic CCK1R activation by ^1^O_2_ after transduction of genetically encoded photosensitizer *in situ* would provide a convenient way to verify unambiguously intrinsic physiological functions of CCK1R in multiple CCK1R-expressing cells or tissues, or to actuate CCK1R function in expressing and non-expressing cell types after transduction with fused KillerRed-CCK1R, miniSOG-CCK1R or other similar constructs.

### Physiological Relevance and Perspectives

The present work found that CCK1R is activated by type II photodynamic action (i.e., ^1^O_2_) irrespective of the photosensitizers used or the CCK1R-expressing cell types. Therefore CCK1R is a unique *G p*rotein *c*oupled *r*eceptor *a*ctivated *b*y *s*inglet *o*xygen (*GPCR-ABSO*). The ^1^O_2_ could in the future be provided *in vivo* by photodynamic action of knocked-in expression of photosensitizers such as KillerRed, miniSOG, to use the *GPCR-ABSO* property to confirm unambiguously CCK1R functions by directing focused light to central or peripheral cells or tissues. ^1^O_2_ can be generated endogenously from photodynamic action in the skin. Skin photodynamic action is triggered after absorption of sunlight in the ultraviolet A region by endogenous photosensitizers. ^1^O_2_ is also generated in neutrophil respiratory burst in neutrophil-infiltrated/inflamed tissues. Therefore the *GPCR-ABSO* property of CCK1R is highly relevant in cellular physiology and is likely to play a significant role in future physiological research.

## Author Contributions

ZJC conceived the idea of the project, supervised all the experiments, and finalized the manuscript. HNJ performed the experiments with KillerRed, YL with miniSOG, and WYJ with SALPC. HJ, YL, and WYJ wrote up the respective sections and all authors checked and approved the final submitted version.

## Conflict of Interest Statement

The authors declare that the research was conducted in the absence of any commercial or financial relationships that could be construed as a potential conflict of interest.

## Supplementary Material

FIGURE S1 Plasmid pKillerRed-CCK1R.
