# Supplementary figures and images for: Cholecystokinin 1 Receptor – A Unique G Protein-Coupled Receptor Activated by Singlet Oxygen (GPCR-ABSO)
Source: Front Physiol. 2018 May 8;9:497. doi: 10.3389/fphys.2018.00497 (PMC5953346; doi:10.3389/fphys.2018.00497)

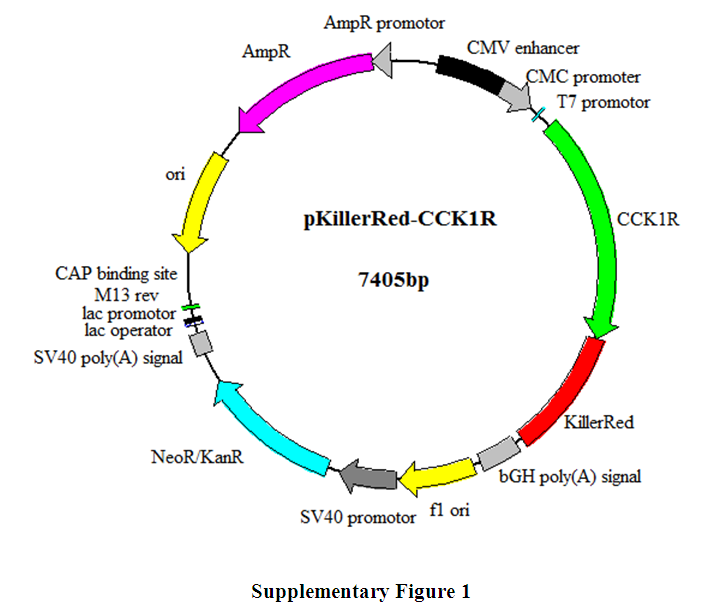

Supplement: FIGURE S1 — Plasmid pKillerRed-CCK1R. [file Image_1.tif]
